# Special Issue: Thermo-Mechanical Behaviour of Structural Lightweight Alloys

**DOI:** 10.3390/ma12152364

**Published:** 2019-07-25

**Authors:** Guillermo Requena

**Affiliations:** 1 Department of Metallic Structures and Hybrid Materials Systems, Institute of Materials Research, German Aerospace Centre, Linder Höhe, 51147 Cologne, Germany; Guillermo.Requena@dlr.de; 2 Metallic Structures and Materials Systems for Aerospace Engineering, RWTH Aachen University, 52062 Aachen, Germany

The need to reduce the ecological footprint of (water, land, air) vehicles in this era of climate change requires pushing the limits in the development of lightweight structures and materials. This requires a thorough understanding of their thermo-mechanical behaviour at several stages of the production chain. Moreover, during service, the response of lightweight alloys under the simultaneous influence of mechanical loads and temperature can determine the lifetime and performance of a multitude of structural components 

The present Special Issue, formed by eight original research articles, is dedicated to disseminating current efforts around the globe aiming at advancing in the understanding of the thermo-mechanical behaviour of structural lightweight alloys under processing or service conditions. The two most prominent families of lightweight metals, namely aluminium and magnesium alloys, are represented with five and three contributions, respectively. 

The work by Poletti et al. [1] deals with the evolution of the microstructure of an AA6082 alloy during thermo-mechanical processing. The production of wrought aluminium alloys usually comprises successive thermo-mechanical steps that involve complex physical phenomena at the microstructural level. Based on flow data and thorough microstructural observations they propose a physically-based constitutive model that can reproduce the behaviour of the alloys during cold and hot working over a wide range of strain rates. The same alloy was studied in [2] by Wiechmann et al. In this case, the authors studied the evolution of the microstructure and the mechanical behaviour of MIG welded joints applying several complementary ex situ and in situ experimental techniques. The results obtained in this work are a step forward to understand the influence of welding heat on the softening behaviour of this alloy. Also dealing with wrought Al alloys, although in a different alloy system, the work by Kowalski et al. [3] investigates the effect of low-temperature thermomechanical treatment (LTTT) on the microstructure, mechanical behaviour and corrosion resistance of a 7000 series AlZn6Mg alloy. Interestingly, they report conditions for LTTT which render better mechanical performance than conventional heat treatments. Moreover, they show that the electrochemical corrosion resistance of the alloy decreases with increasing plastic deformation, while, on the other hand, stress corrosion resistance is improved. 

Bugelnig et al. [4] report on the effect of Ni concentration on the damage accumulation during high temperature tensile deformation of AlSi12Cu4Ni2–3 piston alloys. Using 3D and 4D synchrotron imaging the authors show that interconnecting branches within highly interconnected brittle networks of aluminides determine the damage evolution and ductility in these alloys. A load partition model that considers the loss of interconnecting branches within the rigid networks owing to damage is proposed to rationalize the experimental observations. The last contribution dealing with Al alloys addresses the characterization of A206 (AlCu4.5Mg) wires reinforced with 5 wt% of Al_2_O_3_ nanoparticles. This composite has potential application for TIG welding of aluminium [5]. Here, Florián-Algarín et al. show that a significant strengthening is obtained by the addition of the so-called nanocomposite and that the addition of Al_2_O_3_ affected the electrical conductivity of the wires. 

The contributions on Mg alloys are focused on development, processing and characterization of high strength alloys [6,7,8]. Gavras et al. [6] and Gao et al. [7] explore the improvement of mechanical performance by the addition of rare earth elements. Gavras et al. [6] investigated the evolution of strength and ductility at room and elevated temperature as a function of Nd addition to pure Mg and Mg–Zn alloys. They show that the binary MgNd4 alloy performs better than the ternary alloys up to an addition of 8 wt% of Zn. On the other hand, Gao et al. [7] address the effect of Ce addition on the microstructure of a MgGd7Y3.5Zn alloy. Ce promotes the formation of long period stacking order (LPSO) phases and show that an addition of 0.5 wt% Ce can result in an improvement of mechanical performance. Finally, Garcés et al. [8] present an in-depth report on the effect of severe plastic deformation of Mg-LPSO alloys. This group, which is in one of the pioneers in the study of LPSO-containing Mg alloys, shows that yield strengths similar to extruded conditions can be achieved with only half of the usual Y and Zn contents, owing to the grain refinement provoked by equal channel angular pressing. 

I am confident that the readers will find the contributions to this special issue appealing since they address timely topics to further advance the development of structural Al and Mg alloys. 
